# Bromide alleviates fatty acid‐induced lipid accumulation in mouse primary hepatocytes through the activation of *PPARα* signals

**DOI:** 10.1111/jcmm.14347

**Published:** 2019-04-29

**Authors:** Yujie Shi, Wenxiang Zhang, Yinlong Cheng, Chang Liu, Siyu Chen

**Affiliations:** ^1^ State Key Laboratory of Natural Medicines China Pharmaceutical University Nanjing China; ^2^ School of Life Science and Technology China Pharmaceutical University Nanjing China; ^3^ State Key Laboratory of Pharmaceutical Biotechnology Nanjing University Nanjing China

**Keywords:** bromide, chloride channel, free fatty acids, lipid accumulation, *PPARα*

## Abstract

Increased plasma free fatty acids (FFAs) and liver triglyceride (TG) accumulations have been implicated in the pathogenesis of hepatic steatosis. On the other hand, trace elements function as essential cofactors that are involved in various biochemical processes in mammals, including metabolic homeostasis. Notably, clinical and animal studies suggest that the plasma levels of bromide negatively correlate with those of TG, total cholesterol (TC) and high‐density lipoprotein‐cholesterol (HDL‐C). However, the effect of bromide on lipid accumulation and the direct molecular target responsible for its action remains unknown. Oil red O (ORO) and Nile red staining were used to detect the effect of bromide on lipid accumulation in mouse primary hepatocytes (PHs) treated with different doses of sodium bromide (NaBr) in the presence of FFAs (0.4 mM oleate/palmitic acid 1:1). Spectrophotometric and fluorometric analyses were performed to assess cellular TG concentrations and rates of fatty acid oxidation (FAO), respectively, in mouse PHs. We found that bromide decreased FFA‐induced lipid accumulation and increased FFA‐inhibited oxygen consumptions in mouse PHs in a dose‐dependent manner via activation of *PPARα*. Mechanical studies demonstrated that bromide decreased the phosphorylation levels of JNK. More importantly, the *PPARα*‐specific inhibitor GW6471 partially abolished the beneficial effects of bromide on mouse PHs. Bromide alleviates FFA‐induced excessive lipid storage and increases rates of FAO through the activation of *PPARα*/JNK signals in mouse PHs. Therefore, bromide may serve as a novel drug in the treatment of hepatic steatosis.

## 1 INTRODUCTION

Non‐alcoholic fatty liver disease (NAFLD), which is regarded as the most relevant cause of chronic liver disease, prevails widely in the world.^1^ Epidemiological studies indicate that the prevalence of NAFLD is thought to be approximately 35% in western countries and is clearly related to the growing epidemic of multiple metabolic diseases, such as obesity and cardiovascular diseases.^2^ Thus, NAFLD is a global health crisis that imposes a substantial burden not only on personal health but also on societies and global economies. More importantly, NAFLD may develop into more severe diseases, from non‐alcoholic steatohepatitis (NASH), fibrosis, and cirrhosis to hepatocellular carcinoma.^2^ Therefore, the prevention and treatment of NAFLD is acutely necessary and has great clinical value. Currently, lifestyle changes, dietary alterations and bariatric surgery are common practices for the treatment of hepatic steatosis.^3^, ^4^ A wide range of drugs, including antioxidants, insulin sensitizers, and lipid‐lowering agents, have been applied in clinical trials for the treatment of NAFLD.^5^, ^6^ However, although animal and cellular studies contribute promising advancements, few pharmaceutical therapies exhibit positive outcomes in clinical trials. In this regard, it is urgent to develop novel pharmacological therapies that could ameliorate NAFLD and its related metabolic syndromes.

NAFLD is always associated with hepatic steatosis, which is defined as the abnormal accumulation of triglyceride (TG) over 5% of the liver weight.^7^ Steady state balance of TG in the liver contributes to the pathogenesis of hepatic steatosis, including lipogenesis, lipolysis and fatty acid oxidation (FAO).^8^ Of note, in past decades, trace elements were found to be closely correlated with the progression of NAFLD, as well as with hepatic steatosis. For example, increased liver iron levels are detected in approximately 30% of unselected patients with NAFLD,^9^ whereas an iron‐deficient diet improves insulin sensitivity in patients with NAFLD.^10^ Animal experiments revealed that a marginal iron deficiency diet enhances de novo lipogenesis in rats fed with a sucrose diet.^11^ Moreover, plasma selenium levels are increased with NAFLD risk in humans.^12^ Indeed, selenium exposure increases liver TG concentrations. On the other hand, hepatic copper concentrations are decreased in NAFLD patients and in subjects with other chronic liver diseases.^13^ Animal experiments indicate that a copper‐deficient diet could cause liver steatosis and insulin resistance.^14^ Hence, manipulating concentrations of trace elements in the diet would be a novel strategy in the treatment of NAFLD and hepatic steatosis.

It should be noted that bromide is one of the most abundant and ubiquitous trace elements in the biosphere. In the 20th century, bromide was increasingly introduced into the environment as a salt‐mining waste product and a degradation product of fumigants, leading to the inevitable exposure of bromide for the general population via their food intake.^15^ Therefore, the bromide ion as a residue in food necessitates its broad toxicological and physiological evaluation. Generally, the biological behaviour of bromine is similar to that of chlorine, such that bromide competes with the body chloride and *vice versa*.^16^, ^17^ However, this phenomenon is not obviously validated in the rat thyroid gland, where bromide replaces iodide but not chloride, leading to damage of the thyroid.^15^ Clinically, bromide serves as a sedative for drug production, especially for hypnotic drugs. Additionally, bromide supplementation in dialysate dramatically improves the sleeping quality of patients who are undergoing haemodialysis. Lastly, several clinical investigations suggest that plasma levels of bromide negatively correlate with those of total cholesterol (TC) and high‐density lipoprotein‐cholesterol (HDL‐C).^18^ Brominated vegetable oils reduce the levels of plasma TG, TC and HDL‐C in rats,^19^ suggesting its potential role in the treatment of NAFLD and hepatic steatosis. However, these findings are in the descriptive stage, and the direct molecular target responsible for the beneficial action of bromide remains unknown.

In this study, we used free fatty acid (FFA)‐stimulated mouse primary hepatocytes (PHs) as an in vitro model to examine the effect of bromide on hepatic steatosis. We found that sodium bromide (NaBr) attenuated FFA‐induced excessive fat accumulation and increased FAO through activating *PPARα* signals. Moreover, such an activation effect on *PPARα* is dependent on the chloride channel. Collectively, these findings imply that, in addition to its clinical use in the treatment of epilepsy,^20^ bromide also has great potentials in the prevention and treatment of chronic liver disease related to hepatic steatosis.

## 2 MATERIALS AND METHODS

### 2.1 Cell culture

Mouse PHs were isolated from 6–8‐week‐old mice by using the collagenase II (Sigma, St. Louis, MO, USA) perfusion method, as previously described and were cultured in a humidified atmosphere that contained 5% CO_2_ at 37°C. For bromide treatment, a stock that contained 10 mM NaBr was prepared by using sterile ddH_2_O.

### 2.2 Cell viability assay

CCK‐8 toxicity assay was performed to analyse potential toxic effects of NaBr on cell viability of mouse PHs. Briefly, 10^4^ cells were seeded into each well of a 96‐well plate and were cultured at 37°C overnight. After synchronization with serum‐free DMEM, PHs were transferred into 100 μL serum‐free DMEM containing either NaBr or equal amounts of sodium chloride (NaCl, negative control) at indicated concentrations and incubated for another 24 hours. Then, 10 μL WST‐8 reagent (Jiancheng, Nanjing, China) was added to each well and incubated at 37°C for 2 hours. Finally, a microplate reader was used to measure the absorbance at 450 nm. Cell viability was also analysed by using MTT (Jiancheng, Nanjing, China, 0.2 mg/mL) assay according to the manufacturer's instruction.

### 2.3 Oil red O & Nile red staining

Oil red O (ORO) was purchased from Sigma, St. Louis, MO, USA. In brief, PHs were fixed with 4% paraformaldehyde for 30 minutes and then stained with 0.5% ORO (*w*:*v*) for 15 minutes at room temperature. All the stained sections were examined by light microscopy (400 × magnification). For Nile red (Sigma, St. Louis, MO, USA) staining, PHs were fixed as described previously and were co‐incubated with 0.1 mg/mL Nile red and 10 μg/mL DAPI for 10 minutes. After washing, Nile red‐stained neutral lipids (red) and DAPI‐stained nuclei (blue) were photographed with a Nikon fluorescence microscope (400 × magnification, ECLIPSE, Ts2R‐FL, Tokyo, Japan).

### 2.4 Measurement of intracellular triglyceride

For the intracellular TG content in PHs, cells were lysed by 0.1% Triton X‐100 (Solarbio, Beijing, China) and cell lysates were used to assess the TG content using a commercial kit according to the manufacturer's instructions (Jiancheng, Nanjing, China).

### 2.5 Measurement of fatty acid oxidation

Mouse PHs were seeded and cultured in 96‐well plates overnight (1 × 10^4^ cells per well). For the fatty acid oxidation assay, culture media were replaced by glucose‐deprived DMEM overnight. Oxygen consumption was measured by using an FAO kit (Abcam, Cambridge, MA, USA) and an Extracellular Oxygen Consumption kit (Abcam, Cambridge, MA, USA) according to the manufacturer's instructions.

### 2.6 RT‐qPCR analysis

Total RNA was isolated using Trizol reagent (Invitrogen, Carlsbad, California, USA), reverse transcribed with the PrimeScript RT reagent kit (Takara, Tokyo, Japan) and analysed by real‐time quantitative PCR using 2 × ChamQ Universal SYBR qPCR Master Mix (Vazyme, Nanjing, China) according to the manufacturer's instructions. Primers for mouse *36B4* were used as an internal control. Primer sequences (Table 1) were synthesized by Generay Biotech Co., Ltd. (Shanghai, China).

**Table 1:** List of primer sequences for qPCR analysis

Gene	Primer sequence (5' ‐ 3')
*Srebp‐1c*	Forward: GATCAAAGAGGAGCCAGTGC
*Fasn*	Reverse: TAGATGGTGGCTGCTGAGTG
	Reverse: GAGACGTGTCACTCCTGGACTTG
*Acaca*	Forward: TCAGTCCTGTGTCAGTTTCC
	Reverse: GCTTTTGTTCTCTTCCCG
*Elovl5*	Forward: TGATTTCCCTGATTGCTCT
	Reverse: CTGTTGGTGTGTCCGTTG
*Acly*	Forward: AGGTCTCTCTGCAGCCATGT
	Reverse: AAGCTTTCCTCGACGTTTGA
*Atgl*	Forward: CAACGCCACTCACATCTACG
	Reverse: ACCAGGTTGAAGGAGGGATG
*Hsl*	Forward: CCAGGAATCCTCATTCTGGA
	Reverse: TGGCCAATGGATGTGAAGTA
*Cgi58*	Forward: GTCTAGTGCAGCGTTTGAGGG
	Reverse: ATCTATGCAGGATCGGGCTC
*Pparα*	Forward: ATGCCAGTACTGCCGTTTTC
	Reverse: GGCCTTGACCTTGTTCATGT
*Acox1*	Forward: GCCTGCTGTGTGGGTATGTCATT
	Reverse: GTCATGGGCGGGTGCAT
*Ehhadh*	Forward: CAGATGAAGCACTCAAGCTTG
	Reverse: ACCTTGGCAATGGCTTCTGCA
*Cyp4a10*	Forward: AGCTTGTCAACTTGCCCATG
	Reverse: TGCTGTCCCCATTCTCCATT
*Cpt1a*	Forward: CTCAGTGGGAGCGACTCTTCA
	Reverse: GGCCTCTGTGGTACACGACAA
*36B4*	Forward: GAAACTGCTGCCTCACATCCG
	Reverse: GCTGGCACAGTGACCTCACACG

### 2.7 Western blotting analysis

For protein analysis, cells were lysed in RIPA buffer. The protein concentration was quantified with a BCA Protein Quantitation Assay Kit (Beyotime Biotech., Shanghai, China). Equal amounts of protein were loaded and separated by 10% SDS‐PAGE and then transferred onto a PVDF membrane (Millipore Corp., Billerica, MA, USA). The membranes were incubated overnight with appropriate primary antibodies at 4°C. Bound antibodies were then visualized using horseradish peroxidase‐conjugated secondary antibodies. A quantitative analysis was performed by using ImageJ software (US National Institutes of Health). For the antibody information, the antibody against *PPARα* was purchased from Proteintech (Chicago, IL, USA). Anti‐phospho‐JNK (Thr183/Tyr185), anti‐total JNK, anti‐phospho‐ERK1/2 (Thr202/Tyr204), anti‐total ERK1/2, anti‐total p38, anti‐phospho‐GSK3β (Ser9) and anti‐total GSK3β antibodies were obtained from Cell Signalling Technology (Danvers, MA, USA). The antibody against anti‐phospho‐p38 (Thr180/Tyr182) was purchased from Bioworld Technology, Inc (Nanjing, China). The antibody against GAPDH was derived from Kangcheng Biotech (Shanghai, China).

### 2.8 Statistical analysis

Groups of data were presented as the means ± standard deviation (SD). Data were analysed by using one‐way ANOVA followed by Fisher's LSD *post hoc* test. Calculations were performed by using Origin 8 (version 8.6, OriginLab, Northampton, MA, USA). A value of *P* < 0.05 was considered statistically significant.

## 3 RESULTS

### 3.1 Bromide alleviates free fatty acid induced excessive lipid accumulation in mouse primary hepatocytes

**Figure 1:**
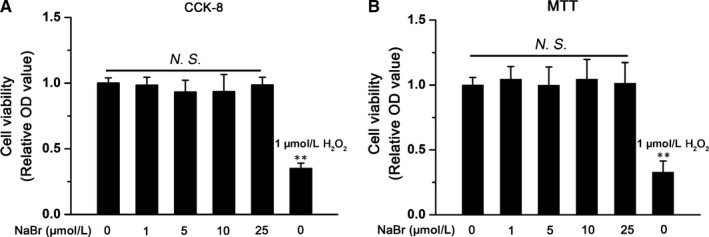
Cell viability analysis. Mouse primary hepatocytes were treated with the indicated doses of NaBr for 24 h. Cell viability was assessed by (A) CCK‐8 and (B) MTT assays. H_2_O_2_ (1 μM) was used as a cytotoxic positive control. CTL: control. *NS* means no significance. **P < 0.01 vs CTL group. All the data were represented as the mean ± SD

**Figure 2:**
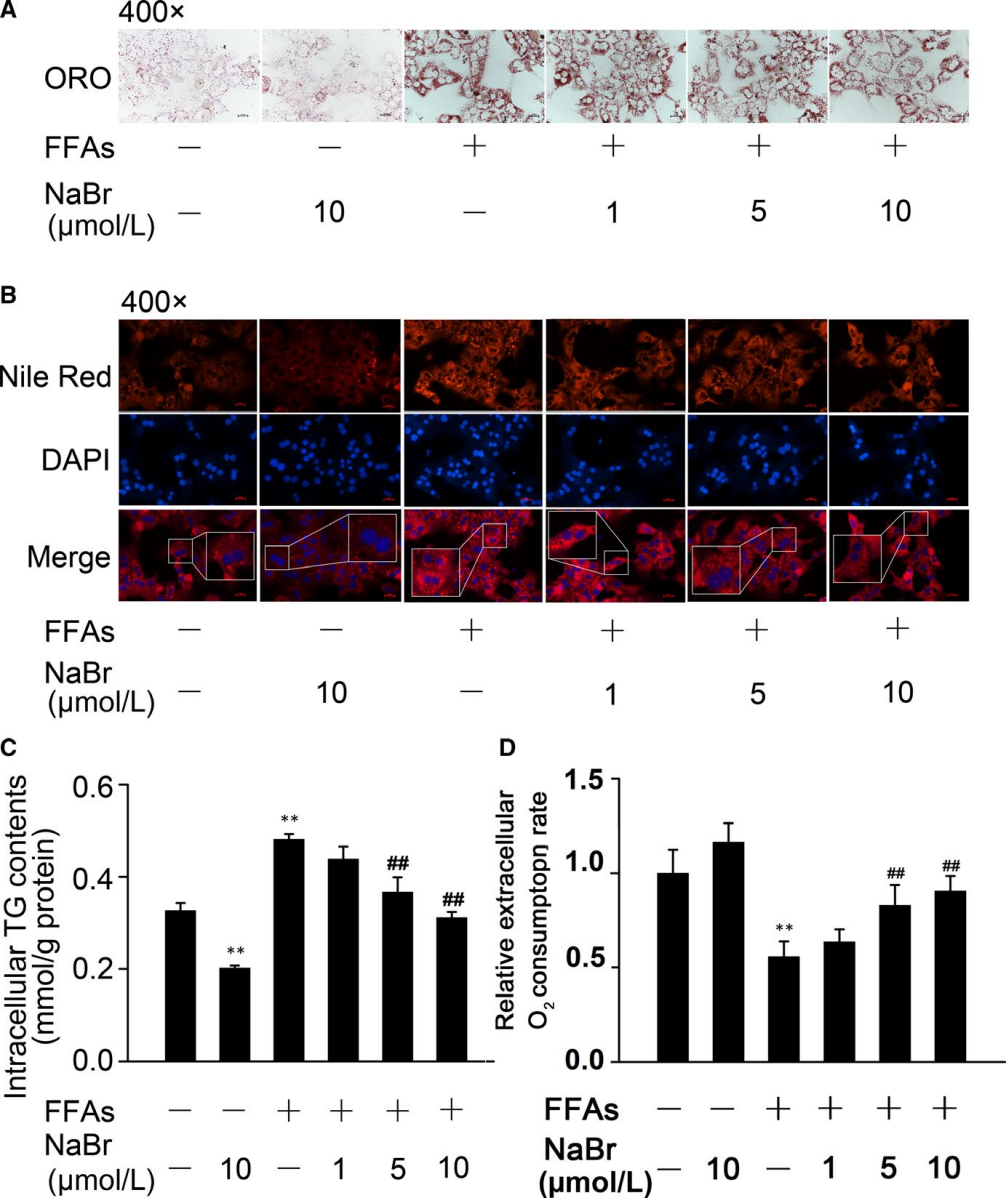
Bromide alleviates free fatty acid (FFA) induced excessive lipid accumulation in mouse primary hepatocytes (PHs). Mouse PHs were incubated with NaBr at indicated doses for 12 h and then treated with 0.4 mM FFAs for 12 h. (A) Oil Red O staining and (B) Nile Red staining. Nile Red, red; nuclei, blue. Scale bar: 10 μm. (C) Intracellular TG contents and (D) fatty acid oxidation. CTL: control. **P < 0.01 vs CTL group; ^##^ *P* < 0.01 vs FFAs group. All the data were represented as the mean ± SD

As shown in Figure 1, both CCK‐8 and MTT incorporation assays demonstrated that while a well‐known cytotoxic agent H_2_O_2_ dramatically decreased the cell viability of mouse PHs, NaBr was not toxic to these cells when its concentration was lower than 25 μM. Therefore, dose ranges from 1 μM to 10 μM were regarded as safe and were chosen for the subsequent experiments. ORO staining revealed that FFAs indeed increased the lipid contents in mouse PHs. In contrast, pre‐treatment of cells with NaBr significantly antagonized the FFA‐induced lipid accumulation in a dose‐dependent manner (Figure 2A). These results were confirmed by Nile red staining in mouse PHs (Figure 2B). Excessive lipid storage is caused by either overactivated lipogenesis (storage) or decreased rates of fatty acid β‐oxidation (consumption). Therefore, we evaluated the cellular concentrations of triglyceride and the rates of fatty acid β‐oxidation. As shown in Figure 2C, FFAs increased the intracellular TG content by ~ 1.5 fold, while NaBr partially attenuated the accumulative effect of FFAs on intracellular triglycerides in mouse PHs. In contrast, 0.4 mM FFAs stimulation dramatically inhibited the oxygen consumption rates of mouse PHs to 56.8%, whereas NaBr reactivated the FAO rates to 90.6% compared to control group (Figure 2D). Notably, all these beneficial effects were in a bromide dose‐dependent manner.

### 3.2 Bromide modulates lipid metabolism genes in mouse primary hepatocytes

**Figure 3:**
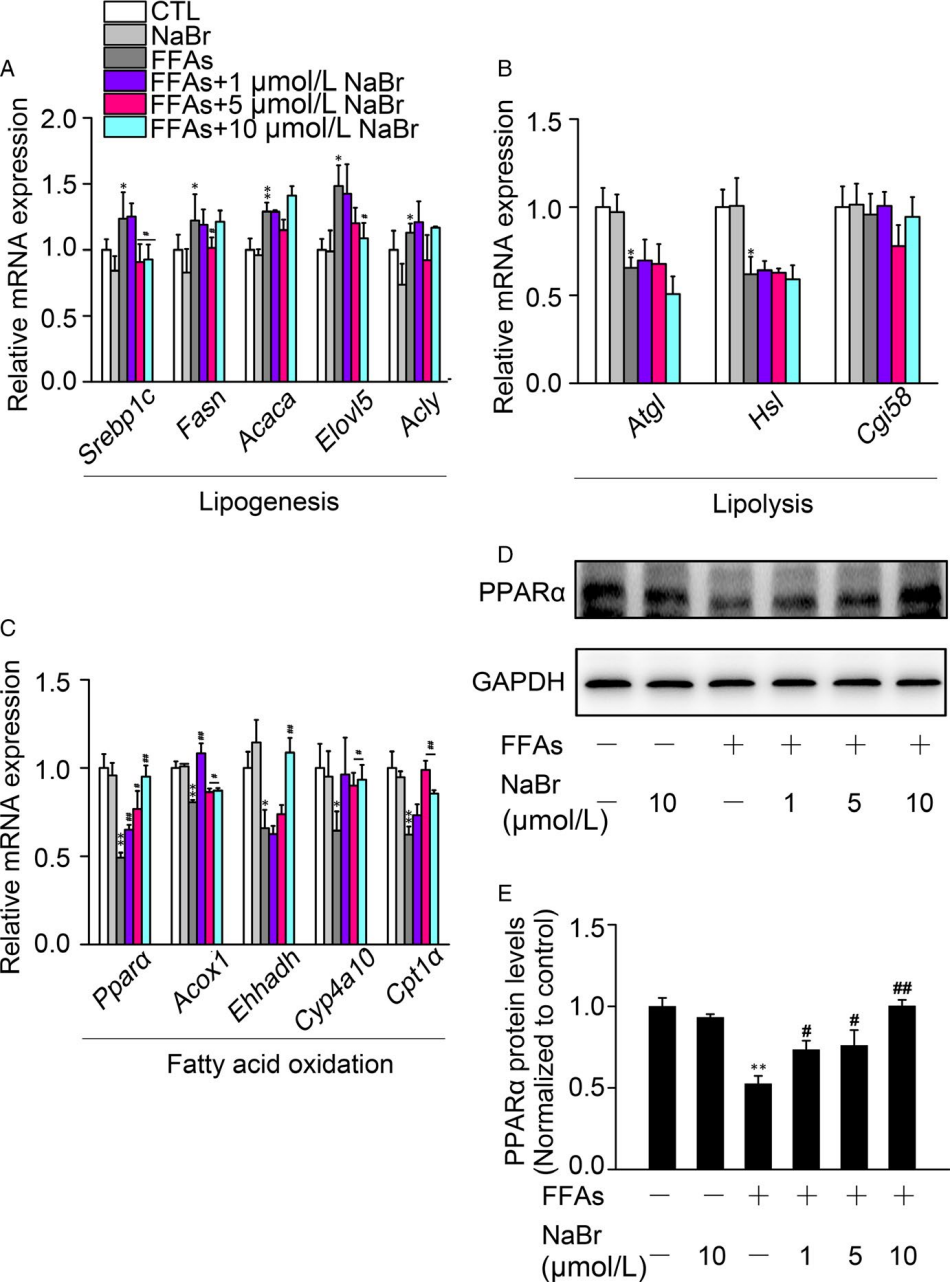
Bromide modulates lipid metabolism genes in mouse primary hepatocytes (PHs). Mouse PHs were treated with NaBr for 12 h and with 0.4 mM free fatty acids (FFAs) for 6 h thereafter. RT‐qPCR analysis determined the hepatic mRNA expression levels of key regulators in lipid metabolism, including (A) lipogenesis, (B) lipolysis and (C) fatty acid oxidation. (D) Western blot analysis of protein expression levels of *PPARα*. (E) Densitometric determinations of (D). CTL: control. *P < 0.05 and **P < 0.01 vs CTL group; ^#^ *P* < 0.05 and ^##^ *P* < 0.01 vs FFAs group. All the data were represented as the mean ± SD

The FFA‐induced lipid accumulation is mainly associated with the following steps: accelerated lipogenesis, impaired lipolysis and retarded FAO. To dissect the molecular events underlying the beneficial effects of NaBr, we used RT‐qPCR analysis to quantify mRNA expression levels of hepatic genes involved in these processes. As shown in Figure 3A, lipogenesis‐related genes promoting synthesis of de novo monounsaturated fatty acids, such as *Srebp1c*, *Fasn*, *Acaca*, *Elovl5* and *Acly*, demonstrated a significant increase in their mRNA expression levels upon FFA stimulation, while only a minor reduction in the *Srebp1c* (by 29.4%) and *Elovl5* (by 26.6%) mRNA occurred upon 10 μM NaBr pre‐treatment. In contrast, FFA administration caused a dramatic reduction in mRNA expression levels of lipolysis‐associated genes, such as *Atgl* (by 34.5%) and *Hsl* (by 38.0%). However, NaBr exhibited a modest effect on their mRNA expression levels (Figure 3B). Furthermore, hepatic mRNA expression levels of *Pparα*, *Acox1*, *Ehhadh*, *Cyp4a10*, and *Cpt1a*, the genes participating in fatty acid β‐oxidation, were decreased upon FFA stimulation. NaBr supplementation recovered the mRNA expression levels of *Pparα* and *Cpt1a* (Figure 3C). More importantly, *Pparα* mRNA levels were positively correlated with NaBr supplementation in response to FFAs and were in a NaBr concentration‐dependent manner. Thus, we have suggested that *PPARα* is the potential drug target of bromide. Consistently, stimulation of mouse PHs with FFAs decreased the protein expression levels of *PPARα* to 51.0% compared to the control group, while pre‐treatment with NaBr dose‐dependently recapitulated the inhibitory effects of FFAs on *PPARα* protein expression (Figure 3D, E).

### 3.3 Bromide decreases the free fatty acid induced lipid accumulation through *PPARα* in mouse primary hepatocytes

**Figure 4:**
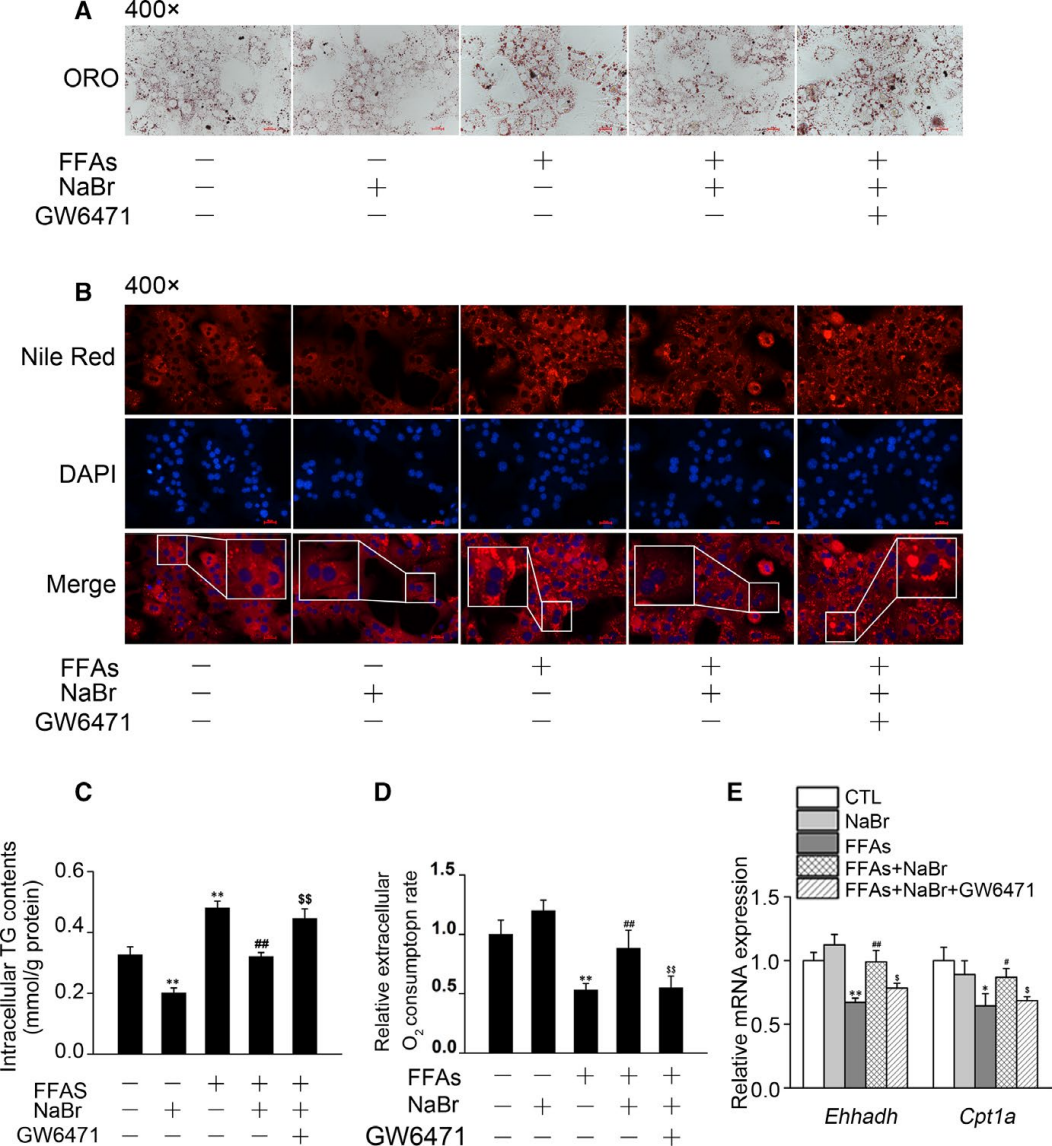
Bromide decreased free fatty acid (FFA) induced lipid accumulation through *PPARα* in mouse primary hepatocytes (PHs). Mouse PHs were pre‐incubated with 10 μM GW6471 for 2 h and then treated with NaBr and FFAs, as previously described. (A) Oil Red O staining and (B) Nile Red staining. Nile Red, red; nuclei, blue. Scale bar: 10 μm. (C) Intracellular TG contents and (D) fatty acid oxidation. (E) RT‐qPCR analysis of mRNA expression levels of *PPARα* target genes. CTL: control. *P < 0.05 and **P < 0.01 vs CTL group; ^#^ *P* < 0.05 and ^##^ *P* < 0.01 vs FFAs group; ^$^ *P* < 0.05 and ^$$^ *P* < 0.01 vs FFAs + NaBr group. All the data were represented as the mean ± SD

Given that *PPARα* is an important nuclear factor that regulates hepatic lipid β‐oxidation, we explored the potential relationships between *PPARα* activation and the alleviation of FFA‐induced lipid accumulation induced by NaBr. To address this issue, we used a *PPARα*‐specific antagonist, GW6471, to quench *PPARα* activity in mouse PHs. As shown in Figure 4A, ORO staining analysis revealed that pre‐treatment with GW6471 partially released the block of FFA‐induced lipid accumulation by NaBr. This result was also confirmed by Nile red staining (Figure 4B). Similarly, pre‐treatment with GW6471 attenuated the inhibitory effects of NaBr on intracellular TG contents (Figure 4C). In addition, GW6471 suppressed the action of NaBr and recovered the activation of FAO (Figure 4D). At the molecular level, *Ehhadh* and *Cpt1a* were two promising *PPARα* downstream targets. RT‐qPCR analysis indicated that NaBr significantly restored the inhibitory effects of FFAs on the mRNA expression levels of *Ehhadh* (by 47.3%) and *Cpt1a* (by 34.9%). However, GW6471 partially abrogated the restoration effects of NaBr on mRNA expression levels of these two genes (Figure 4E).

### 3.4 Bromide inhibits free fatty acids induced phosphorylation of JNK in mouse primary hepatocytes

**Figure 5:**
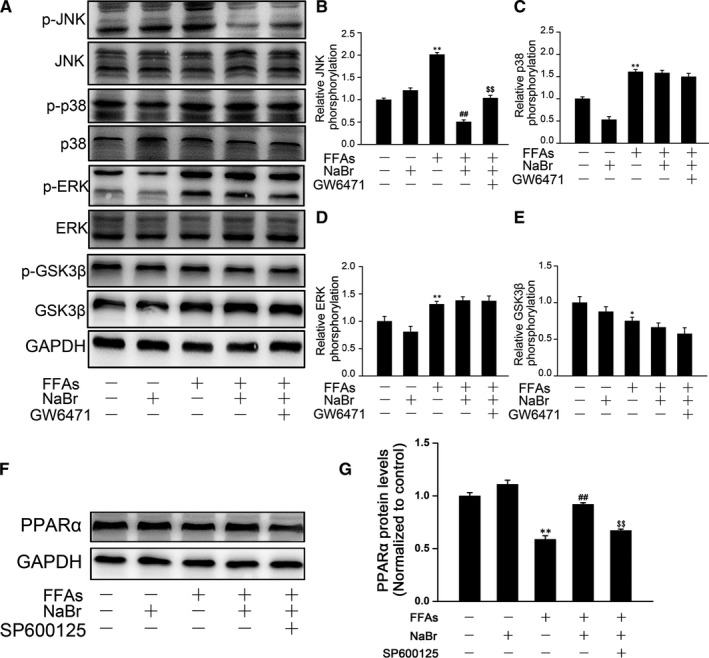
Effect of bromide on phosphorylation levels of JNK, ERK and p38. (A‐E) Mouse primary hepatocytes (PHs) were treated with 10 μM NaBr for 24 h and then stimulated with 0.4 mM free fatty acids (FFAs) for 30 min. GW6471 was added to the cells for 2 h in advance when required. Western blot and densitometric determination analyses of phosphorylation levels of JNK, ERK, p38 and GSK3β. (F) Mouse PHs were pre‐treated with SP600125 (10 μM) for 2 h and then treated with NaBr and FFAs, as previously described in Figure 3. Western blot analysis of protein expression levels of *PPARα*. (G) Densitometric determinations of (F). CTL: control. *P < 0.05 and **P < 0.01 vs CTL group; ^##^ *P* < 0.01 vs FFAs group; ^$$^ *P* < 0.01 vs FFAs + NaBr group. All the data were represented as the mean ± SD

The mitogen‐activated protein kinases (MAPKs, including JNK, ERK and p38 MAPK) have been proposed to play critical roles in maintaining lipid homeostasis.^21^, ^22^, ^23^ Therefore, to delineate the cellular and molecular mechanisms underlying bromide‐induced alleviation of FFA‐triggered excessive lipid accumulation in mouse PHs, we evaluated the effect of bromide on the phosphorylation of MAPK signalling cascades. As shown in Figure 5A‐D, phosphorylated JNK, ERK2, and p38 MAPK were increased upon 12 hours stimulation with FFAs. In contrast, pre‐treatment with NaBr markedly reduced the phosphorylation levels of JNK, while it exhibited modest effects on the ERK and p38 MAPK phosphorylation. Once again, GW6471 partially dampened the NaBr‐induced inhibition of JNK phosphorylation (Figure 5A, B). Furthermore, the phosphorylation levels of the GSK‐3β were decreased upon FFA stimulation, suggesting this key mental disease‐associated factor 24 was indeed involved in the pathogenesis of hepatic steatosis. However, NaBr treatment did not rescue the activity of GSK‐3β (Figure 5A, E).

While NaBr activates *PPARα* and inhibits JNK phosphorylation, it is interesting to evaluate the role of JNK pathway in the bromide‐induced *PPARα* expression. As shown in Figure 5F, G, the specific JNK inhibitor, SP600125, partially diminished the increase in *PPARα* protein expression induced by bromide, suggesting that JNK is a dominant signalling pathway mediating bromide‐induced *PPARα* up‐regulation.

### 3.5 Bromide represses free fatty acids induced excessive lipid accumulation and restores *PPARα* expression through the chloride channel

**Figure 6:**
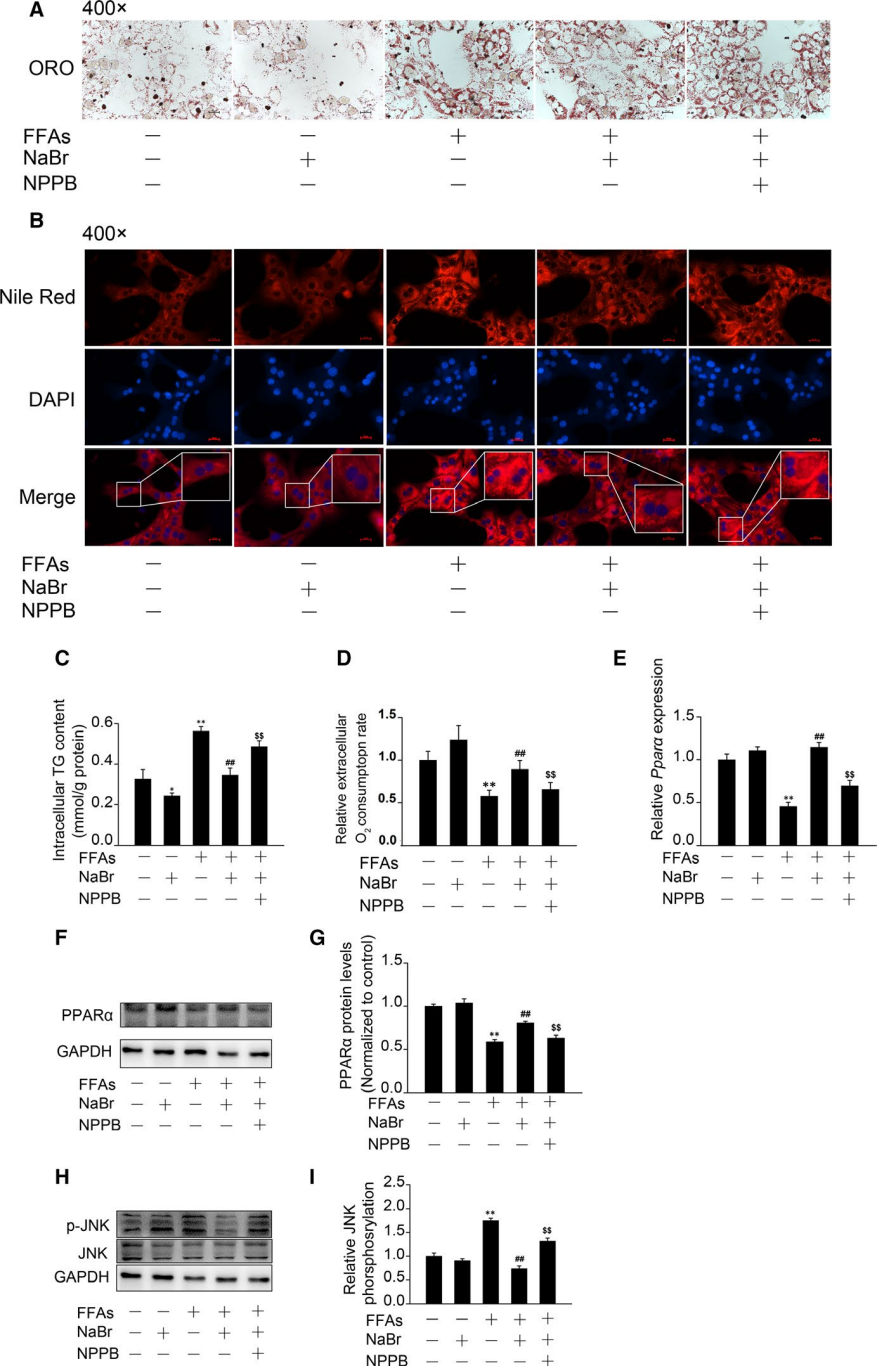
Bromide represses free fatty acid (FFA) induced excessive lipid accumulation and restores *PPARα* expression through the chloride channel. Mouse primary hepatocytes (PHs) were treated with 10 μM NaBr for 12 h and then stimulated with 0.4 mM FFAs for 12 h. NPPB was added to the cells for 1 h in advance when required. (A) Oil Red O staining and (B) Nile Red staining. Nile Red, red; nuclei, blue. Scale bar: 10 μm. (C) Intracellular TG contents and (D) fatty acid oxidation. (E‐G) mRNA and protein expression levels of *PPARα*. Mouse PHs were treated as describe in Figure 5. NOTE: NPPB was added to the cells for 1 h in advance when required. (H & I) Western blot and densitometric determination analyses of phosphorylation levels of JNK. CTL: control. *P < 0.05 and **P < 0.01 vs CTL group; ^##^ *P* < 0.01 vs FFAs group; ^$$^ *P* < 0.01 vs FFAs + NaBr group. All the data were represented as the mean ± SD

Given that bromide is able to penetrate the cell membrane through chloride channels in the neuron system,^25^ we examined whether bromide possessed these beneficial functions through the chloride channel in mouse PHs. As shown in Figure 6A, B, pre‐treatment with 5‐nitro‐2‐(3‐phenylpropylamino)‐benzoic acid (NPPB, a potent inhibitor of the chloride channel) abolished the preventive effect of NaBr on FFA‐induced lipid accumulation. In addition, NPPB treatment recovered the inhibition of intracellular TG contents induced by NaBr in response to FFA stimulation (recovered to ~ 1.4 folds, Figure 6C). Once again, NPPB reduced the oxygen consumption rates to 81.7% as compared to the NaBr and FFA co‐treated group (Figure 6D). More importantly, NPPB diminished the NaBr‐restored *PPARα* expression and recovered the NaBr‐repressed JNK phosphorylation (Figure 6E‐I).

## 4 DISCUSSION

**Figure 7:**
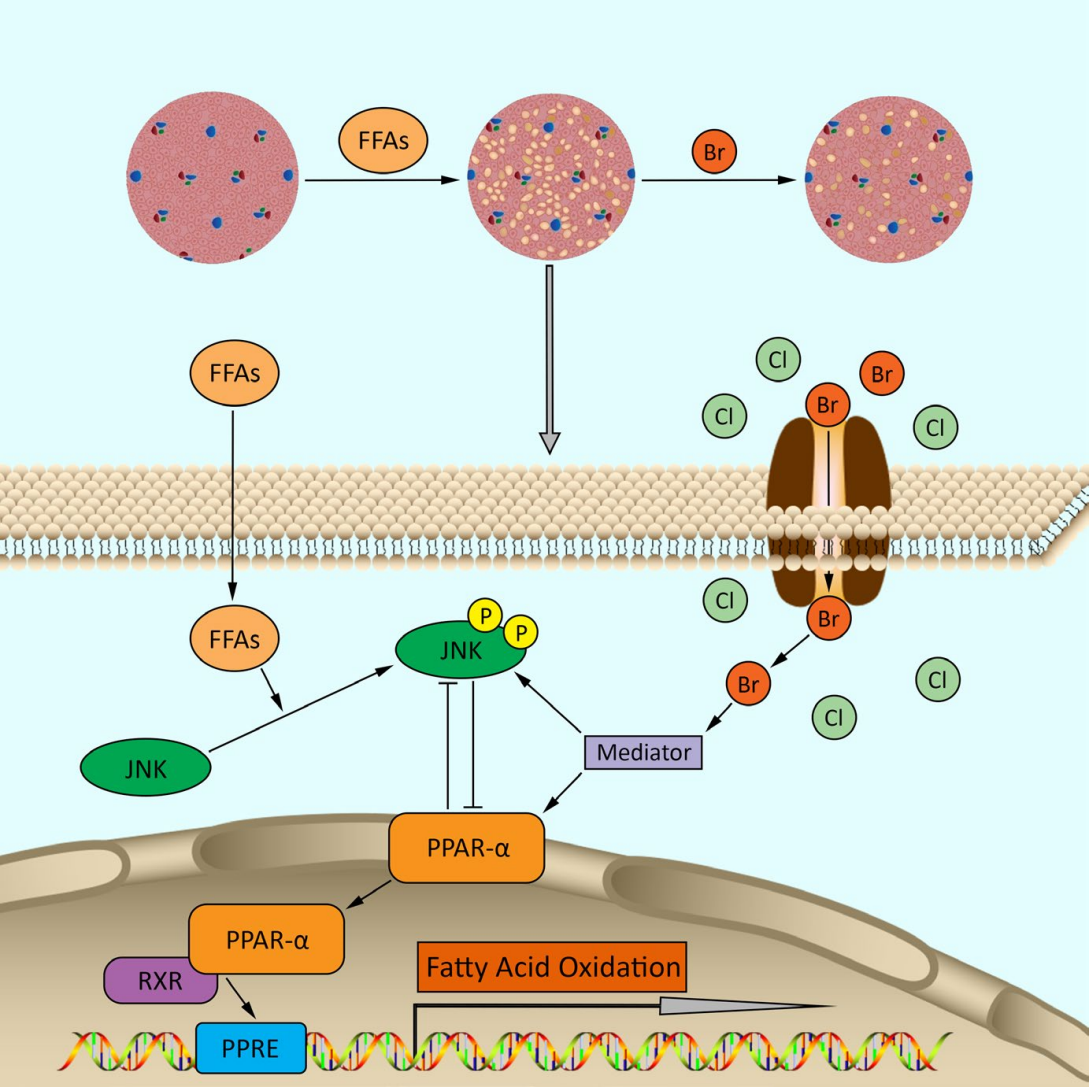
The functional model illustrating the mechanism by which bromide decreases the free fatty acid induced fat accumulation in mouse primary hepatocytes, highlighting the mediating role of *PPARα*/JNK pathways in the bromide signal relay

New drug development is always established through two major methods: (a) Drug discovery: bring new medicines to the market. However, several disadvantages exist, including the long and expensive R&D cycle and the toxic failures of newly developed drugs upon clinical application^26^; (b) Drug reposition: reapplying existing drugs to new medical conditions. This could shorten the R&D process to 3‐12 years and accelerate phase I‐III clinical trials.^26^, ^27^ The applications of drug repositioning have indeed led to successful remedies for various medical conditions, including cancer and cardiovascular diseases. In our previous study, we found a new strategy for a mood stabilizer (lithium chloride) in alleviating balloon‐induced neointimal hyperplasia.^28^ As an old drug, bromide has been used to treated epilepsy for a long time.^20^ In the 1990s, clinical and experimental studies indicated the negative correlations between bromide and plasma lipids (including TG, TC and HDL‐C).^18^ However, the effects of bromide on lipid metabolism in the liver and the direct molecular target in mediating such effects remain unknown. In this study, we focused on the protective effects of bromide on lipid dysregulation and found that NaBr repressed FFA‐induced lipid accumulation while increasing FAO (Figure 2), as well as the phosphorylation of JNK in mouse PHs (Figure 5A, B). Of note, using GW6471, a *PPARα*‐specific inhibitor, partially recapitulated the beneficial effects of NaBr (Figure 4). In addition, a chloride channel blocker, NPPB, attenuated the NaBr‐induced activation of *PPARα* expression (Figure 6E‐G). Therefore, we propose that the hypolipidemic function of NaBr is dependent on the activation of *PPARα* and signal relay of the chloride channel (Figure 7). Collectively, our results provide a new function for bromide in the prevention and treatment of hepatic steatosis and its related metabolic diseases such as NAFLD, thus providing strong evidence for the drug repositioning of bromide.

Lipid homeostasis is dependent on the balance between lipogenesis (storage), lipolysis and fatty acid β‐oxidation (consumption).^29^ Among these, lipogenesis is controlled by a cluster of transcriptional factors, such as SREBP1c,^30^ which drive their targeting genes (eg ELOVL5).^31^ Transcriptional factors such as *PPARα* regulate the expression of genes involved in mitochondrial and hepatic fatty acid β‐oxidation.^32^ In this study, we found that FFAs increased the mRNA expression levels of SREBP1c and FASN (Figure 3A) but decreased the levels of hepatic *PPARα* and CPT1a mRNA transcripts in mouse PHs, while NaBr treatment showed the opposite effects (Figure 3C). More importantly, the recapitulation of *PPARα* expression at both mRNA and protein levels was in a bromide dose‐dependent manner (Figure 3C‐E). Thus, we concluded that *PPARα* might be the target of bromide, which was confirmed by the antagonism effect of GW6471 pre‐treatment (Figure 4). How this activation occurs remains a subject of further investigations including structural or physiological binding analysis. On the other hand, drugs targeting *PPARα*, fibrates, have been developed and used in the clinical application for a long time.^33^ Although these fibrates possess satisfactory properties in managing the lipid homeostasis; most of them have adverse or side effects. For example, fibrate uptake causes nausea, stomach upset, and sometimes diarrhoea.^34^, ^35^ Long‐term administration of fibrates impairs liver function and causes muscle damage.^36^ Notably, as an essential trace element, bromide plays a pivotal role in maintaining redox homeostasis at a serum concentration of 42 ~ 61 μM in healthy individuals.^18^, ^37^ Therefore, the doses we selected were in a tolerable range to humans and were much lower than those used in the clinic (~2.1 mM). Such a low dose of bromide will dramatically decrease the toxic crisis and improve patient tolerance in future applications. Further studies need to be conducted to evaluate the lipid‐lowering effects and toxicity of bromide in vivo based on these low doses.

Chloride ions are the most common anions in living organisms, participating in numerous biological progresses through transmembrane transport and anion channels. At the hepatocyte plasma membrane, chloride channels are essential for cell volume control and apoptosis regulation.^38^, ^39^ More importantly, mice with cystic fibrosis transmembrane conductance regulator (CFTR, a chloride channel) deficiency exhibit a remarkable impairment of PPARγ function in both colonic epithelial cells and the lung, indicating the causal relationship between chloride channel and the PPAR family.^40^, ^41^ Here, we found that the activating effect of NaBr on *PPARα* signals was partially, if not totally, abrogated by the chloride channel blocker NPPB, suggesting the potential regulatory roles of the chloride channel in pathways downstream of *PPARα*, such as FAO (Figure 6). Additionally, as an ion channel located on the cell membrane, the chloride channel is more easily manipulated than *PPARα*, which may lead to more specific regulation of lipid metabolism. On the other hand, decreased activity of the chloride channel in vascular smooth muscle cells positively correlates with the development of hypertension. Our findings indicated that pre‐treatment of NPPB repressed the lipid‐lowering effects of NaBr. Therefore, the inhibition of chloride channel actions may be consistently harmful for health. As NAFLD is related to an increased risk of hypertension,^42^ activation of this ion channel may provide a therapeutic option to prevent the further deterioration of hepatic lipid control and cardiovascular functions.

The mitogen‐activated protein kinases (MAPKs, including JNK, ERK and p38 MAPK) have been proposed to play critical roles in maintaining lipid homeostasis. For example, a high‐fat diet causes JNK activation in the liver, further activating SREBP‐induced lipogenesis.^21^ In contrast, phosphorylation of p38 MAPK triggers hepatic lipid oxidation through activation of *PPARα*.^23^ In our study, FFA stimulation indeed increased phosphorylated JNK, ERK and p38 MAPK levels, whereas NaBr treatment specifically antagonized JNK phosphorylation (Figure 5A‐F). It should be noted that JNK activation could phosphorylate *PPARα*,^43^ thus inhibiting the FAO process. However, we found that GW6471 abolished the inhibition effect of NaBr on JNK phosphorylation (Figure 5A, B). This result implies an upstream role of *PPARα* on JNK phosphorylation, which has been similarly observed in other studies. For example, the *PPARα* agonist Wy‐14643 has been shown to decrease acetaminophen‐induced JNK phosphorylation in mouse livers, whereas *PPARα* deficiency abolished this inhibition effect.^44^ Similarly, another agonist of *PPARα*, fenofibrate, protects the kidney from hypertensive injury in spontaneously hypertensive rats via inhibition of JNK phosphorylation.^45^ Hence, *PPARα* and JNK may form a feedback loop in the regulation of the hepatic FAO process. Further investigations will be necessary to elucidate the causal relationship between these two molecules. In addition, such an inhibition effect of NaBr was abolished by pre‐treatment with NPPB (Figure 6H, I). Collectively, these data suggest: 1) p‐JNK activity may serve as the molecular basis for the hypolipidemic effects of NaBr; 2) *PPARα* induction partially mediates the NaBr‐induced dephosphorylation of JNK; 3) Chloride channels dominantly, if not totally, participate in the NaBr‐induced activation of the *PPARα*/JNK axis. On the other hand, as an important drug target, GSK‐3β increases the phosphorylated tau protein, which is an essential factor in epilepsy. Our results showed that FFA stimulation caused the rapid activation of GSK‐3β (dephosphorylation) in mouse PHs. However, NaBr pre‐treatment did not retard GSK‐3β activation (Figure 5G, H). The modest effect of NaBr on the dephosphorylation of GSK‐3β indicated the doses of NaBr, ranging from 1 to 10 μM, are relatively safe for the nervous system.

In conclusion, this study demonstrates that NaBr alleviates FFA‐induced excessive lipid storage and increases the rate of FAO through the activation of the *PPARα*/JNK pathways and the signal relay of the chloride channel in mouse PHs (Figure 7). These findings imply that in addition to its improvement ability in epilepsy, bromide is also a promising candidate for the prevention and treatment of hepatic steatosis.

## 5 DATA AVAIBILITY STATEMENT

The data that support the findings of this study are available from the corresponding author upon reasonable request.

## CONFLICT OF INTEREST

All authors declare that they have no competing interest.
